# Beta-HCG Secretion by a Pulmonary Choriocarcinoma in a Male Patient

**DOI:** 10.1155/2024/8731806

**Published:** 2024-01-27

**Authors:** Brecht Devos, Cedric Willemse, Mira Sterckx, Johan Debruyne, Inge Stappaerts, Tom Van den Mooter, Marjan Hertoghs, Pascale Abrams

**Affiliations:** ^1^GZA Hospitals Antwerp, Department of Urology, Wilrijk, Antwerp, Belgium; ^2^GZA Hospitals Antwerp, Department of Endocrinology, Wilrijk, Antwerp, Belgium; ^3^GZA Hospitals Antwerp, Department of Pneumology, Wilrijk, Antwerp, Belgium; ^4^GZA Hospitals Antwerp, Department of Oncology, Wilrijk, Antwerp, Belgium; ^5^GZA Hospitals Antwerp, Department of Anatomical Pathology, Wilrijk, Antwerp, Belgium

## Abstract

**Background:**

Paraneoplastic secretion of beta-subunit of human chorionic gonadotropin (*β*-HCG) in pulmonary carcinoma is rare. *Case Presentation*. A 65-year-old man presented with bilateral gynaecomastia with abnormally high levels of *β*-hCG and elevated oestradiol, progesterone, and testosterone levels on April 7, 2023. After excluding testicular malignancy, CT scan of the chest and abdomen revealed bilateral pulmonary lesions. Transthoracic biopsy confirmed malignancy with choriocarcinoma. MRI of the brain showed a solitary brain metastasis, while on a subsequent 18F-FDG PET/CT, no other metastatic lesions were seen. The patient was treated with chemoimmunotherapy carboplatin-etoposide-pembrolizumab with good partial response.

**Conclusion:**

Our case of a presumably stage IV dedifferentiated mNSCLC presenting as an extragonadal *β*-hCG secreting pulmonary choriocarcinoma is a very rare tumor with a poor prognosis. Its biology, origin, and treatment remain to be elucidated. Cancer genome sequencing is necessary for the identification of the origin and seeking treatment.

## 1. Introduction

Choriocarcinoma is a rare germ cell tumor that shows abnormally high levels of *β*-human chorionic gonadotropin (*β*-hCG), mainly in women in intrauterine pregnancy. Primary choriocarcinoma of the gonads is known to exhibit rapid hematogenous spread, commonly to the lungs, but also to the bone, liver, brain, and spleen. It is scarce to occur outside the gonads. Extragonadal primary choriocarcinomas occur most commonly in the body's midline in the retroperitoneum, mediastinum, pineal gland, or the middle of the cranium [1]. We report a male patient who was diagnosed with a very rare entity of a presumably dedifferentiated metastatic non-small-cell lung cancer (mNSCLC) presenting as an extragonadal pulmonary choriocarcinoma with bilateral lung masses and a brain metastasis, NSCLC stage IV. We provide our clinical, imaging, immunohistochemistry (IHC), and genetic findings in the hope to improve the accuracy of the diagnosis and provide new insights into the pathogenesis of the disease.

## 2. Case Presentation

**Figure 1:**
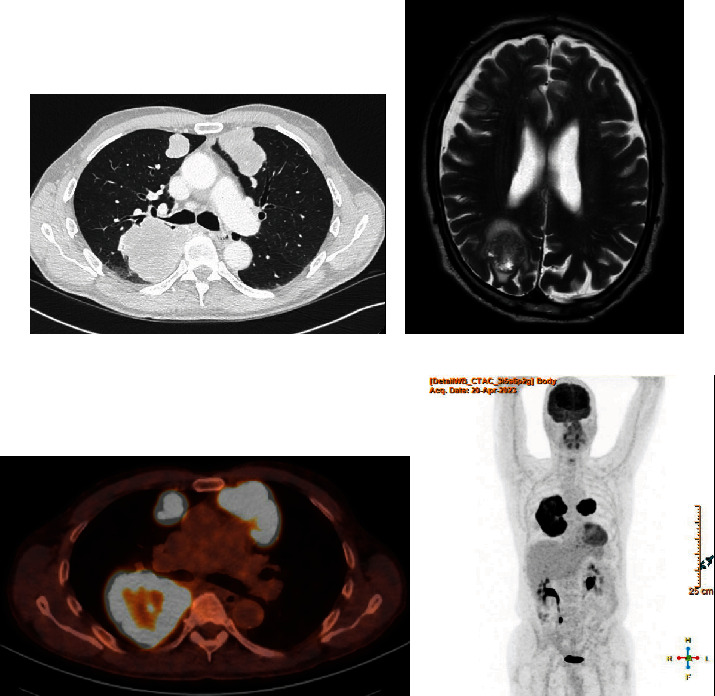
(a) A computed tomographic scan of the chest showing the 3 pulmonary masses with the largest mass located posteriorly in the right lower lobe. (b) MRI of the brain (T2-weighted axial image) showing the solitary brain metastasis consisting of a 2.7 cm nodular component with perilesional edema and hemorrhagic characteristics: susceptibility artifacts with foci of signal loss on T2-gradient echo sequence in the peripheral part of the nodule. (c, d) 18F-FDG PET/CT showing FDG avidity and extent of the bilateral pulmonary tumoral sites with physiologic uptake in the urinary tract.

A 65-year-old male presented for evaluation of bilateral gynaecomastia, loss of libido, agitation, and cough since 5 months. He had a medical history of alcohol abuse requiring multiple admissions to psychiatry since 2015 and a 50 pack-year history of smoking. Eastern Cooperative Oncology Group (ECOG) performance status is 0. Physical examination revealed no testicular masses and no clinically enlarged lymph nodes, including supraclavicular palpation. Chest examination showed bilateral vesicular breath sounds. Labs were significantly high for *β*-hCG levels of 8.16 U/L (Ref < 0.10 U/L) and HCG levels of 491 U/L (Ref < 6 U/L) and elevated for oestradiol of 126 ng/L (Ref 11-44 ng/L), progesterone of 1.3 *μ*g/L (Ref 0.1-0.2 *μ*g/L), and testosterone levels of 44.5 nmol/L (Ref 7.66–24.82 nmol/L), i.e., hypergonadotropic hypergonadism. Other tumor markers that were measured (alpha-fetoprotein, neuron specific enolase, and carcinoembryonic antigen) were within normal limits. A scrotal ultrasound could not reveal any testicular or intrascrotal lesion suspicious for malignancy. The high *β*-hCG prompted the initial suspicion of and further investigations for an extragonadal germ cell tumor. A computed tomography (CT) scan of the thorax, abdomen, and pelvis was performed which disclosed bilateral pulmonary masses (*n* = 3) arising from the lung parenchyma with the greatest mass measuring 12 cm in the right lower lobe (Figure 1). An MRI of the brain revealed a solitary metastasis in the right parieto-occipital region with a nodular component of 2.7 cm and perilesional edema. 18F-FDG PET/CT showed no other metastatic lesions. Two CT-guided transthoracic core needle biopsies of the lung mass in the right lower lobe were subsequently performed.

**Figure 2:**
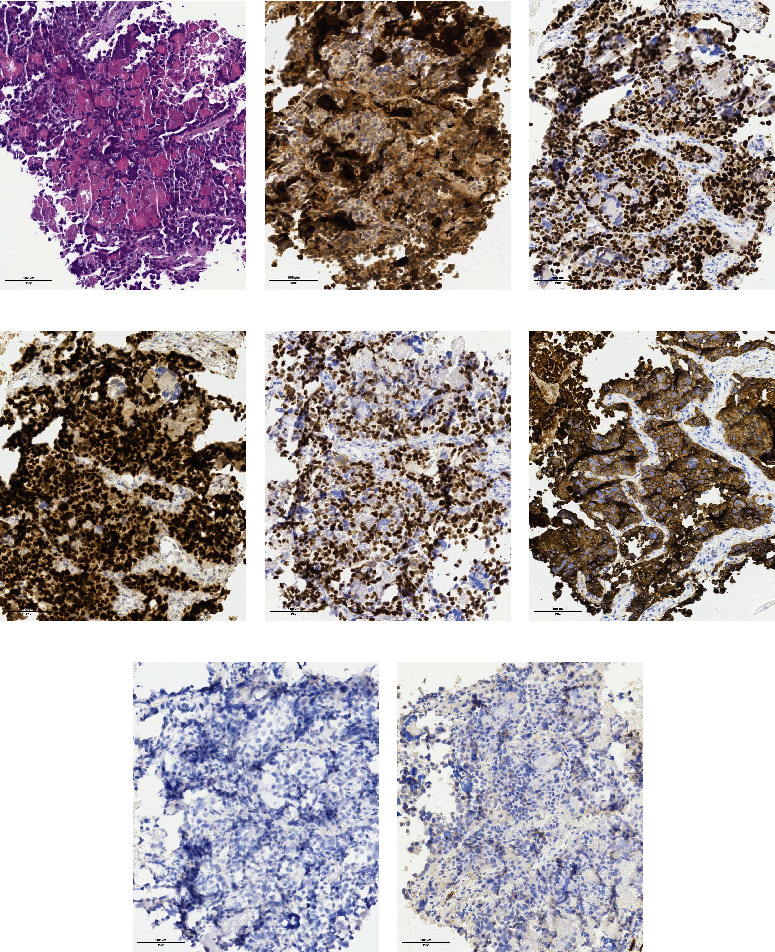
Histologic and IHC findings. (a) Malignant tumor with extensive necrosis, mostly composed of large tumor cells with enlarged hyperchromatic nuclei and eosinophilic to clear cytoplasm (hematoxylin and eosin stain, ×20). (b) HCG is immunopositive in the cytoplasm of tumor cells (×20). (c) SALL-4 is positive in the nucleus of most tumor cells (×20). (d) GATA-3 is firmly nucleus positive in mononuclear trophoblast cells (×20). (e) P40 are nucleus positive in mononuclear trophoblast cells (×20). (f) CK pan is diffused and strongly positive in the cytoplasm of tumor cells (×20). (g) TTF-1 IHC is negative in the tumor cells (×20). (h) D2-40 is negative in the tumor cells (×20). HCG: human chorionic gonadotropin; GATA-3: anti-GATA3 antibody; CK pan: pankeratin; TTF-1: thyroid transcription factor 1.

Microscopic pathological result is malignant with extensive tumor necrosis (Figure 2). IHC results are as follows: HCG(+), SALL4(+), GATA3(+), p40(+), pankeratin (CK pan)(+), thyroid transcription factor 1 (TTF-1)(-), and D2-40(-). Next-generation sequencing with Oncomine™ focus assay on 35 clinically relevant genes for NSCLC showed no pathogenic mutations, including no EGFR driver mutation or fusion transcript with Idylla™ gene fusion assay. The tumor has programmed cell death-ligand protein 1 (PD-L1) expression with a tumor proportion score (TPS) of 10%.

**Figure 3:**
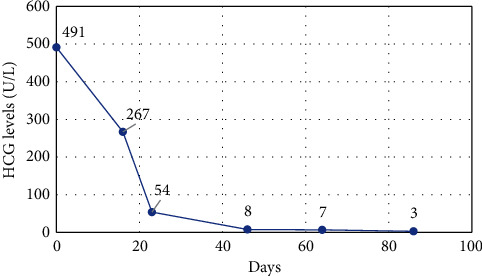
Human chorionic gonadotropin (HCG) levels. Day 0 represents the start of the chemoimmunotherapy.

After discussion by our multidisciplinary team including medical oncologist, pneumologist, radiation oncologist, and radiologist, a combination regimen of chemotherapy and immunotherapy was adopted: carboplatin, etoposide, and pembrolizumab. He successfully completed 4 cycles of carboplatin-etoposide (carboplatin AUC 5 on day 1, every 21 days; etoposide 200 mg on day 1, every 21 days) and pembrolizumab (200 mg on day 1, every 21 days). His *β*-hCG decreased to normal values 3 months after the initiation of chemoimmunotherapy (Figure 3). Follow-up CT scans showed good partial response to therapy of the soft-tissue metastases according to the Response Evaluation Criteria in Solid Tumors version 1.1 (RECIST v1.1) with a ≥30% decrease of the sum of the longest diameter of the pulmonary masses and brain metastasis [2]. The patient was subsequently offered radiosurgery of the solitary brain metastasis using 3 × 8 Gy, but he refused this therapeutic modality.

## 3. Discussion

Extragonadal pulmonary choriocarcinoma is an extremely rare and highly malignant germ cell neoplasm that is commonly fatal. Several theories might be offered for this occurrence of choriocarcinoma in the lung: origin from retained primordial germ cells that migrated abnormally during embryonic development and metastasis from a primary gonadal tumor that regressed spontaneously; another theory is that primary lung carcinomas differentiate into trophoblasts or choriocarcinomas by dedifferentiation [3]. Although rare, some histological types of pulmonary carcinoma express *β*-HCG, such as adenocarcinomas, squamous cell carcinomas, or large cell carcinomas [4]. In the present case, both the possibilities of nongestational choriocarcinoma of the lung and a trophoblastic differentiation from lung parenchymal tissue could not be completely ruled out. Given the discordant clinical findings of the older age of our patient who is an active smoker, more suggesting a pulmonary origin of the tumor, and on the other side the IHC profile of germ cell origin (SALL4+, GATA3+, and TTF-1-), we assumed a dedifferentiation of a NSCLC into choriocarcinoma. A potential limitation in our report is the fact that a definite histopathological diagnosis is best made on a fully resected tumor and not on biopsies, to demonstrate a mixture of components of primary lung carcinoma (adenocarcinoma, etc.) and choriocarcinoma [5]. Zhang et al. recently reviewed the literature, and from 1953 to 2021, 41 male patients with “primary” pulmonary choriocarcinoma were reported [6]. The rarity of extragonadal pulmonary choriocarcinoma results in a lack of standard therapy and late diagnosis. Its anaplastic nature makes extragonadal pulmonary choriocarcinoma recalcitrant to treatment, which also contributes to the high mortality rate with a median survival time of 8 months [7].

Although no clear correlation is seen between smoking and choriocarcinoma mortality, smoking is a well-known risk factor for NSCLC [8]. The effects of alcohol are often confounded by smoking. From a pooled analysis of 7 prospective studies with 399,767 participants and 3137 lung cancer cases, a slightly greater risk of lung cancer was indicated among people who consumed at least 30 g per day of alcohol than among those who abstained from alcohol [9].

The treatment strategy for a patient with newly diagnosed mNSCLC without an oncogenic driver includes consideration of histology, tumor genotype, PD-L1 expression, comorbidities, and the patient's preference. In our patient, we used a combination of platinum-based chemotherapy, which is also suitable for choriocarcinoma treatment, and programmed cell death protein 1 (PD-1)/PD-L1 blockade, which is the most common treatment approach according to the European Society for Medical Oncology (ESMO) 2023 clinical practice guidelines [10]. At the time of manuscript preparation, the patient continues to receive pembrolizumab immunotherapy and still maintains an ongoing good clinical and iconographic response.

## 4. Conclusion

We present a case of *β*-HCG secretion in pulmonary carcinoma, presumably clinically stage IV dedifferentiated NSCLC, presenting as an extragonadal pulmonary choriocarcinoma with solitary brain metastasis. It is a very rare tumor with a poor prognosis. Its biology, origin, and treatment remain to be elucidated. Cancer genome sequencing is necessary for the identification of the origin and seeking treatment to improve survival rates.

## Data Availability

Data sharing is not applicable to this article as no datasets were generated or analyzed during the current study.

## Abbreviations

*β*-hCG: *β*-Human chorionic gonadotropin
CT: Computed tomography
ECOG: Eastern Cooperative Oncology Group
ESMO: European Society for Medical Oncology
IHC: Immunohistochemistry
mNSCLC: Metastatic non-small-cell lung cancer
18F-FDG PET/CT: Positron emission tomography with 2-deoxy-2-[fluorine-18]fluoro-D-glucose integrated with computed tomography
PDL-1: Programmed cell death-ligand protein 1
PD-1: Programmed cell death protein 1
TPS: Tumor proportion score.
